# Sticks *and* carrots: encouraging open science at its source

**DOI:** 10.1002/geo2.2

**Published:** 2015-03-23

**Authors:** Sabina Leonelli, Daniel Spichtinger, Barbara Prainsack

**Affiliations:** ^1^ Exeter Centre for the Study of the Life Sciences and Department of Sociology, Philosophy and AnthropologyUniversity of ExeterExeter; ^2^ European CommissionDG Research and Innovation, Unit A.6. – Science Policy, Foresight and DataBrusselsBelgium; ^3^ Department of Social Science, Health and MedicineKing's College LondonLondon

**Keywords:** open science, open access, incentives, research cultures, research policy

## Abstract

The open science (OS) movement has been seen as an important facilitator for public participation in science. This has been underpinned by the assumption that widespread and free access to research outputs leads to (1) better and more efficient science, (2) economic growth, in particular for small and medium‐sized enterprises wishing to capitalise on research findings, and (3) increased transparency of knowledge production and its outcomes. The latter in particular could function as a catalyst for public participation and engagement. Whether OS is likely to help realise these benefits, however, will depend on the emergence of systemic incentives for scientists to utilise OS in a meaningful manner. While in some areas, the environmental sciences have a long tradition of open ethos, citizen inclusion and global collaborations, such activities need to be more systematically supported and promoted by funders and learned societies in order to improve scientific research and public participation.

## Abstract 2

Reflexion: Does the logic of the University sector allow space for Open Science? A response to Leonelli et al.

Response to Leonelli et al (2015): Thinking About “Open” Science

## Introduction

Open science (OS) has been seen as an important factor in facilitating and catalysing public participation in science. Increasing parts of the material that used to be inaccessible but for professional experts are now accessible to wider groups of people. Open access to scientific peer‐reviewed publications has led this trend, which is now also expanding to original research data. There are, however, still a number of obstacles to OS, which currently hinder the full realisation of its benefits.

At least in principle, OS involves the public dissemination of all elements involved in scientific inquiry, ranging from lab journals and research notes to publications, materials, data, methods/protocols, models, code and software. While not all of these elements may be freely available in all cases, a commitment to facilitate the sharing of these materials underpins the OS movement. This commitment is seen to play a central role in enabling researchers to effectively reuse existing outputs for their own purposes (Royal Society 2012), and to foster the intelligibility and reproducibility of research findings across disciplinary boundaries. It also makes it possible for researchers to pick up and continue research that was started, but never completed, by others (Levin *et al*. submitted). Finally, it is expected to enhance recognition for the efforts involved in producing research components other than journal publications, which could in turn enhance impact and citations for whoever develops such components (Piwowar *et al.* 2007), and to encourage the use of high standards in research, e.g. careful data production, well tested modelling and robust software (Nature Special 2013).

At the same time, increasing transparency in research practices can have unintended consequences. Anything that is open to public scrutiny can be used to assess the practices in question, which may be premature for ongoing projects that need time to yield clear and widely intelligible results. It may also compound researchers’ fears of being scooped. It is not hard to imagine that researchers forced to render lab or field notes, protocols or software freely accessible to others will feel the need to create shadow procedures and infrastructures for those parts of their practice that they do not want, or cannot share (Tenopir *et al*. 2011; Poline *et al.* 2012; Schäfer *et al*. 2011). Finding ways to decide *how* sharing and transparency can be organised to be as fruitful as possible is one of the main challenges at present. However consensus that we need to share seems to have become solid.

Robust funder requirements – for example, as implemented in the US (Office of Science and Technology Policy 2013) and in the EU's Multiannual Research Framework Programme ‘Horizon 2020’ (European Commission 2013a) – are key to a widespread uptake of OS. So far, however, such mandates pertain mainly to access to *publications*. As part of the changing *modus operandi* of the research system, search engines are replacing discipline‐specific journals as the first point of access for most scientists looking for relevant research published by others. It has become anachronistic to address the publication of research findings in scholarly journals as a separate concern from processes through which research is actually conducted: all elements of scientific inquiry, and their different roles in specific phases of research, need to be taken into account. The European Commission, for one, has already started thinking about other means of facilitating OS. It sees OS, sometimes also referred to as ‘Science 2.0' or ‘Science in transition', as helpful in addressing the Grand Challenges of our times, such as demographic change, climate change, health, food security, clean energy and others mentioned within Horizon 2020 (European Commission 2013b). The notion of ‘Science 2.0’ signifies that every aspect of scientific practice is currently undergoing changes (European Commission 2014). Examples for such changes are the emergence of alternative reputation systems, the growing use of scientific blogs, open annotation, and widening access to data and publications. ‘Science 2.0’ as a *holistic approach* thus includes much more than Open Access. It represents a paradigm shift in the *modus operandi* of research and science spanning the entire scientific research cycle, from the inception of research to its publication and future use. It also affects the evaluation of the quality and impact of research. For these reasons, the European Commission is conducting a stakeholder consultation on the issue, including an online public consultation (closed September 2014) whose results are currently being analysed.

### A change in research culture

At the same time, a more widespread uptake of OS requires the support and understanding of researchers on the ground: if OS is perceived by researchers primarily as another piece of bureaucracy imposed by funders, compliance will be a best half‐hearted. In addition to the ‘stick’ of compulsory mandates, ‘carrots’ are therefore also needed. This can only be achieved by changes in the scientific culture at large. In particular the following systemic shifts are needed:

1. *Recognition of sharing practices in credit structures.* Meaningful sharing takes time; data that are just dumped into a repository without sufficient metadata, annotations or other relevant information may meet the sharing requirements imposed by some funders or universities, but the chances that the data will be discoverable and usable by others are very low. Thus, effort and time that researchers invest in the meaningful sharing of data, protocols, notes and results need to be considered in career progression decisions (e.g. promotion, tenure, etc.) and research assessments. Metrics to aid this process need to be developed and implemented (Bourne 2014). OS is, by definition, not a solipsistic activity but a community effort. If assessment metrics for scientific researchers took into consideration the contribution that they make to facilitate the free flow of information and ideas within the scientific community as well as within society as a whole, this would be a strong incentive for people who would like to support OS but cannot afford to (because they, for example, need to focus on activities that will get them tenure instead).
2. *Creation of more meaningful incentives for researchers to engage with OS*. A current obstacle to a wider uptake of OS is that many researchers know very little about the variety of formats of OS and their consequences. For example, research institutions should provide systematic training to scientific researchers on practices such as self‐archiving, on different formats of data sharing and its advantages and potential downsides (in the medical domain in particular, where an important concern is the possible re‐identification of individuals), or on how to make information intelligible for specific intended user groups. Moreover, as an increasing number of institutions run research or teaching initiatives around ‘big data’, it is important that these are not narrowly focused on technical skills such as predictive analytics or data cleaning, but they deal with big data comprehensively, including its societal, ethical, philosophical and regulatory aspects. This will lead to higher levels of awareness of the potential benefits and drawbacks of OS among scientific researchers as well as among wider publics, which in turn facilitates more meaningful and targeted support of OS. Also within the more limited area of open access to published findings, we have not reached the end of the road. Better incentives to engage with open access publishing of research findings will need to be created in order to demonstrate to researchers that OS can be a way to reach audiences and users more effectively and that will be beneficial to researchers themselves, for instance as concerns their citation rate (Caball *et al.* 2013). The use of social media is important, but by far not the only aspect of this. As incentives are likely to vary depending on the specific features of each research area, both discipline‐specific research funders and learned societies have an important role to play in promoting meaningful engagement with OS within their disciplines. We already mentioned the need for medical professionals and others working with personal information to be trained in a kind of ‘social impact assessment’ of sharing research data. Given the discipline‐specific nature of the nature and uses of data themselves, learned societies may be better placed to train researchers on modalities of data sharing than universities or funders, as they would be able to develop and provide specific guidelines addressing the concerns and context characteristic of each field.
3. Recognition of the role of alternative metrics (‘altmetrics’) and changing publication cultures. The emergence of data journals and citable repositories, which incentivise the acknowledgement of data production as a research outcome in itself, is one step in this direction, although it is unclear which role such tools will play in future research assessment exercises. Initiatives promoting the publication of models and protocols have lagged behind in comparison to data sharing tools, and the situation is even worse when it comes to materials such as specimens or cell cultures, whose standardisation and dissemination have mostly been achieved through the open ethos and efforts of specific individuals and communities (when they were achieved at all) (Leonelli and Ankeny 2012; Leonelli 2013). Altmetrics provide a potential solution to this issue, even if currently available altmetrics focus more on the resonance of research in social media than on the extent to which authors make the materials and data relating to their publications freely available. Given the considerable effort involved in disseminating research components, universities and funding agencies should ensure that researchers receive targeted support for conducting these activities, for instance in the form of dedicated funding and additional personnel devoted specifically to managing information sharing. Additionally, an important aspect is to ensure the citability of data, in order to ensure that data creators are properly acknowledged (Kotarski *et al.* 2012).

Such strategies should be at the centre of the next wave of policymaking on OS. Policymakers have already started to realise some of these suggestions. For example, in a 2012 Recommendation the European Commission clearly outlined the need for systemic change (European Commission 2012), suggesting that Member States should ‘adjust the recruitment and career evaluation system for researchers and the evaluation system for awarding research grants to researchers so that those who participate in the culture of sharing results of their research are rewarded’. The Recommendation also states that if researchers make their findings available through open access, this should be taken into account in relevant assessment procedures. The Commission also encourages the use of new and alternative models of assessing and measuring careers and research activities more generally, especially those that encompass not only the publication of research findings but also data and other types of output.

While the European Commission will continue to support these actions – for instance through a call in the 2014–15 ‘Science with and for Society’ Work Programme, the bulk of the changes need to be implemented at the level of EU Member States, or even at the sub‐member states level (depending on the respective research system of the Member State in question). Here, cooperation in the framework of the European Research Area (ERA), which contains an item on ‘improving knowledge circulation’, is potentially very valuable, since it involves both member states and stakeholder groups, such as various associations of European Research Organisations (LERU, Science Europe, EUA, NORDFORSK, CESAR and EARTO). Currently existing approaches that go in the right direction, such as the mandate of the University of Liege for publications, should be explored further and could potentially be supported as ‘best practice’ examples. At the same time, adequate training and support needs to be provided to researchers, so that they are aware, first, of what OS entails and what the potential benefits and concerns are, and second, they can support OS without having to deal with additional administrative burdens. While some EU support exists, such as the OpenAIRE and FOSTER projects, research‐performing organisations will have a major role to play in providing this support. This could take the form of dedicated information managers and could also involve research libraries. Learned societies also need to take responsibility for helping researchers to identify appropriate infrastructures, publication opportunities and relevant tools that may inform both the planning and the dissemination of their work. In parallel to this development, calls for patients or research participants to be given access to the data that are held about them in clinical and research repositories or within medical devices are becoming more frequent (Lunshof *et al*. 2014; Dockser Marcus and Weaver 2012).

### OS in the environmental and life sciences

Within the environmental and life sciences, research communities involved in long‐term longitudinal environmental studies and model organism research have long cultivated an ethos of data sharing and open communication, including efforts to disseminate results on a global scale and to include amateur scientists into research initiatives and publications (e.g. Leonelli and Ankeny 2012; The Long Term Ecological Research Network 2014). These practices emerged in a relatively hostile environment thanks to the perseverance and vision of specific groups of individuals and funding initiatives, and were developed in response to the specific challenges of the research areas in which they emerged, as well as the social and political context in which they were situated. In this sense, they constitute a role model for future research in other areas, and need to be systematically supported and widely publicised by relevant learned societies and funding bodies. This is particularly important in relation to geography, where the modalities and extent of dissemination of results varies enormously across projects and between the physical and human realms, and constructive debate across such research contexts would be highly beneficial to the development of the field as a whole. Physical geography has a long history of data‐heavy research and effective data sharing, particularly in areas such as cartography and oceanography (OceanDataPortal 2014; Martin *et al.* 2009). Nevertheless, the field as a whole has yet to exploit the vast opportunities offered by recent technological and institutional shifts (Kitchin 2013) – a situation partly due to the disciplinary politics underlying large‐scale data collection efforts during and after the Cold War (when geology, climatology and environmental science commanded most data initiatives of potential relevance to geographers), and the extent to which commercial and military institutions have taken ownership of such efforts (e.g. Aronova *et al.* 2010; Aronova[Link]). The same can be said about human geography, where data are typically more sensitive than those collected within physical geography, and researchers need to exercise particular care both in data collection and in the evaluation of which data can be openly disseminated and how (Elwood *et al*. 2012). Establishing networks to discuss modes of data sharing and reflect on their implications could be a productive way to bring physical and human geography into closer dialogue with each other, and enable researchers in these areas to learn and profit from each others’ skillset (such as experience in handling large datasets in the case of physical geography, and in protecting the confidentiality of data on human subjects in the case of human geography).

The IT revolution – which started several decades ago – continues to reverberate in the scientific system. In order to reap the benefits of OS it needs to be implemented in an institutional context that creates incentives for researchers to share and reuse data, addresses transparency concerns and provides adequate support structures. Merely telling researchers to engage and to learn how to use social media is not in the spirit of a kind of OS that will help public engagement.
